# Supporting Birthing People and Supporting Doulas: The Impact of the COVID-19 Pandemic on a Community-Based Doula Organization in San Francisco

**DOI:** 10.1089/heq.2022.0194

**Published:** 2023-06-13

**Authors:** Ashley Nguyen, Stephanie Arteaga, Marlee-I Mystic, Alli Cuentos, Marna Armstead, Jennet Arcara, Andrea V. Jackson, Cassondra Marshall, Anu Manchikanti Gomez

**Affiliations:** ^1^ School of Public Health, University of California, Berkeley, Berkeley, California, USA.; ^2^ Sexual Health and Reproductive Equity Program, School of Social Welfare, University of California, Berkeley, Berkeley, California, USA.; ^3^ SisterWeb San Francisco Community Doula Network, San Francisco, California, USA.; ^4^ Department of Obstetrics, Gynecology and Family Planning, Maternal, Child, and Adolescent Health Program, University of California, San Francisco, San Francisco, California, USA.

**Keywords:** community doula, COVID-19, maternal health services, community network, mental health, public health, ethical issue

## Abstract

**Background:**

Beginning in March 2020, health care systems in the United States restricted the number of support people who could be present during pregnancy-related care to reduce the spread of COVID-19. We aimed to describe how SisterWeb, a community-based doula organization that employs Black, Pacific Islander, and Latinx doulas in San Francisco, California, adapted to the COVID-19 pandemic.

**Methods:**

As part of process and outcome evaluations conducted through an academic-community partnership, we interviewed SisterWeb doulas, mentors, and leaders in 2020, 2021, and 2022 (*n*=26 interviews). We identified preliminary themes using the Rapid Assessment Process and then conducted thematic analysis of data related to COVID-19.

**Results:**

SisterWeb leadership remained committed to safeguarding doulas by shifting to virtual support until doulas were onboarded as benefitted employees. Doulas reported hospital policies impacted clients' pregnancy-related care. Initially, doulas adapted to virtual support by connecting with clients more frequently through phone and text. When permitted to meet in person, doulas adjusted to client preference. Finally, as the pandemic impacted doulas' well-being, they turned to mentors for emotional support.

**Discussion and Health Equity Implications:**

This analysis contributes to a growing body of literature describing doulas' experiences during the pandemic. By shifting to virtual support, SisterWeb leaders prioritized the health, safety, and financial stability of doulas, who were members of communities disproportionately impacted by COVID-19. Our findings suggest that public health guidance, organizational COVID-19 precautions, and hospital policies hindered SisterWeb's goal of ensuring clients receive equitable medical care. In addition, we found that emotional support for doulas is vital to their work.

## Introduction

The COVID-19 pandemic severely impacted people's pregnancy and childbirth experiences in the United States.^1^ To reduce the spread of COVID-19, many health care systems restricted the number of support people who could be present during pregnancy-related appointments and births, and some hospitals did not allow doulas to attend births in person.^2–5^ Doulas are nonclinical birth workers who provide physical, emotional, and informational support before, during, and after childbirth, with well-documented benefits.^6–8^ Community doulas support birthing people at elevated risk of adverse outcomes due to structural racism, usually at no- or low-cost.^9^

Although some doulas shifted to supporting clients virtually during the pandemic, this limited community doulas' ability to bear witness to and mitigate obstetric violence, particularly for clients of color who experience racism and bias in medical settings.^10–14^ Notably, disparities related to COVID-19 and birth outcomes intersect due to institutional racism and structural inequities. In San Francisco, California, Black and Pacific Islander communities are disproportionately impacted by COVID-19^15^ and adverse birth outcomes.^16^

Founded in 2018, the SisterWeb San Francisco Community Doula Network provides culturally congruent community doula care to Black, Pacific Islander, and Latina/o/x families in San Francisco, with the mission of advancing birth equity and providing sustainable dignified employment for community doulas.^17^ Before the pandemic, SisterWeb doulas offered in-person support to all clients throughout its standard course of care (i.e., three prenatal visits, support during labor and birth, and four postpartum visits).^17^ In March 2020, SisterWeb transitioned from in-person to virtual support for births and prenatal and postpartum visits.^18^ Owing to public health orders, hospitals where SisterWeb clients gave birth initially limited the number of support people.^4,19^ In previous studies, doulas described their experiences during the pandemic,^4,10–12,20–23^ but to our knowledge, only one study examined how COVID-19 disrupted community-based doula organizations.^24^ In this study, we describe how SisterWeb adapted to the COVID-19 pandemic.

## Methods

### Case description

SisterWeb officially launched in 2019 with three programs: Kindred Birth Companions (KBC), which pairs Black clients and doulas; M.A.N.A. Pasefika, which pairs Pacific Islander clients and doulas; and Semilla Sagrada, which pairs Latinx clients and doulas.^17^ Within each program, doulas work in cohorts of two to three doulas. Each client is assigned a cohort, allowing doulas to share client responsibilities and work in shifts during long births.^17^ Doulas also receive one-on-one and group mentorship from doula mentors, experienced birth workers who provide professional and emotional support, guidance, and coaching.^17^

In pursuit of SisterWeb's mission to dismantle racist health care systems, the organization developed the Champion Dyad Initiative to create opportunities for bidirectional feedback between doulas and health care providers at birth sites, including hospitals, where SisterWeb clients deliver.^17^ Each birth site has one or two staff “champions” who communicate with SisterWeb staff members during monthly meetings and as issues arise.^17^

### Study design

As part of an academic-community partnership (described elsewhere^25,26^), SisterWeb and university-based researchers have collaborated to design and conduct process and outcome evaluations of two of its three programs, KBC and M.A.N.A. Pasefika. The process evaluation, conducted from 2018 to 2020, focused on whether the programs were implemented as designed, working well, accessible to birthing people and community doulas, and supported by hospital staff. During the process evaluation, we conducted in-depth interviews with clients, KBC and M.A.N.A. Pasefika doulas, doula mentors, organizational leaders, and external partners.

Findings from the process evaluation informed the outcome evaluation, conducted from 2020 to 2022. The outcome evaluation focused on the impact of doula care on clients, doulas, and hospital environments. We also aimed to understand the impact of working with SisterWeb on doulas and mentors and gain their perspectives on the impact of SisterWeb's programs. We conducted additional in-depth interviews with KBC and M.A.N.A. Pasefika doulas and mentors.

For all interviews, we invited potential interviewees to participate through e-mail. During the process evaluation, all KBC and M.A.N.A. doulas, mentors, and leaders were eligible to participate. During the outcome evaluation, KBC and M.A.N.A. Pasefika doulas who had seen clients during the previous year were eligible, as well as mentors who had been on staff during the same time frame. We invited nine SisterWeb doulas to participate in interviews in February–May 2020, eight in February–March 2021, and eight in February–March 2022. We invited four SisterWeb mentors to participate in interviews in January–May 2020 and six in February–March 2021.

Finally, we invited four members of SisterWeb's leadership circle, which oversees program implementation and administration and acts as SisterWeb's decision-making body, to participate in interviews in July 2020. Although we tailored interview guides to each group's scope of work, all interviews explored participants' experiences with and perceptions of program implementation. All interviews were conducted through phone or videoconference, audio-recorded, and professionally transcribed. All participants provided informed consent. The Committee for the Protection of Human Subjects at the University of California, Berkeley approved the study protocol.

Table 1 provides an overview of participants, by year interviewed. We interviewed six doulas in 2020 and 2021 and five doulas in 2022. Three doulas participated in interviews at all three timepoints, three at two timepoints, and two at one timepoint. Two and five mentors agreed to be interviewed in 2020 and 2021, respectively. All four SisterWeb leaders agreed to be interviewed in 2020.

**Table 1.** Participants, By Year Interviewed

Interview participants	***n***
2020 interviews (*n*=12)	
Community doulas	6
Kindred birth companions^a^	4
M.A.N.A. Pasefika^a^	2
Leaders	4
Doula mentors^b^	2
2021 interviews (*n*=11)	
Community doulas	6
Kindred birth companions	4
M.A.N.A. Pasefika	2
Doula mentors	5
2022 interviews (*n*=5)	
Community doulas	5
Kindred Birth Companions	4
M.A.N.A. Pasefika	1

^a^ SisterWeb community doulas were assigned to one of three programs: Kindred Birth Companions paired Black clients and doulas, M.A.N.A. Pasefika paired Pacific Islander clients and doulas, and Semilla Segrada paired Latinx clients and doulas.

^b^ Owing to lack of data related to COVID-19, we excluded the 2020 mentor interviews from our thematic analysis.

### Data analysis

For the analysis, we initially utilized the Rapid Assessment Process (RAP).^27^ For all interviews conducted in 2020 and 2021, we developed a summary template with neutral domains matched to interview questions, which we used to create a summary of each interview. We transferred summaries to an individual-level data matrix to synthesize data across participants and domains. Finally, we created group-level memos to synthesize findings and to identify preliminary themes, including those related to COVID-19, which informed the 2022 doula interview guide. For 2022 interviews, we repeated the RAP steps, with the exception of creating a group-level memo. Owing to lack of data related to COVID-19, we excluded the 2020 mentor interviews from the next phase of our analysis, which draws on 26 interviews total.

Next, we conducted a thematic analysis of data related to SisterWeb's adaptation to the COVID-19 pandemic.^28^ Using Dedoose, an online mixed methods analysis program,^29^ A.N. took an open coding approach to code COVID-19-related data in the 2021 doula and mentor transcripts and develop a codebook. A.N. then coded the 2020 and 2022 transcripts. Using the coded data and preliminary themes identified during the RAP, A.N. developed themes in consultation with A.M.G. Then, all authors but A.V.J. and C.M. met to discuss preliminary themes. We clarified any questions about organizational processes with SisterWeb leadership.

## Results

Our findings reflect how COVID-19 impacted SisterWeb and its mission using the perspectives and experiences of SisterWeb leaders (*n*=4), mentors (*n*=5), and doulas (*n*=8). See Table 2 for participant characteristics. We identified four themes that describe organizational adjustments due to COVID-19: adapting the organization to respond to COVID-19, adapting to changing hospital policies, adapting to shifting modes of client support, and adapting to supporting doulas' well-being.

**Table 2.** Participant Characteristics

	*n* (%)
Community doulas (*n*=8)	
Highest level of educational attainment	
Some college, no degree	2 (25.0)
Associate's degree	1 (12.5)
Bachelor's degree	5 (62.5)
Age, average (range)	
2020 interviews	29.0 (23–38)
2021 interviews	28.3 (22–39)
2022 interviews	28.8 (23–34)
Leaders (*n*=4)	
Highest level of educational attainment	
Associate's degree	1 (25.0)
Bachelor's degree	2 (50.0)
Master's degree	1 (25.0)
Age, average (range)	
2020 interviews	48.8 (42–56)
Doula mentors (*n*=5)	
Highest level of educational attainment	
Bachelor's degree	3 (60.0)
Master's degree	2 (40.0)
Practicing midwife	3 (60.0)
Age, average (range)	
2021 interviews	42.8 (33–57)

### Adapting the organization to respond to COVID-19

As COVID-19 spread, SisterWeb's leadership remained committed to serving clients and safeguarding SisterWeb doulas. SisterWeb also responded to increased client need, as clients could not easily access essential items due to financial challenges, supply shortages, and the risk of contracting COVID-19. Through community partnerships, SisterWeb procured and delivered groceries, baby items, and personal protective equipment (PPE) to clients. Even when hospitals began permitting doulas to attend births in person, SisterWeb chose to continue virtual support until they could transition doulas from independent contractors paid on a per-client basis to hourly employees (up to 32 h a week) with health insurance and sick leave, a process that took several months and is described elsewhere.^25^

Meanwhile, the broader doula community was fighting to attend hospital-based births. Once doulas were permitted to attend in-person births again, SisterWeb leaders faced an “ethical, moral dilemma” (Leader) about whether to send SisterWeb doulas back into hospitals:

“All of our doulas identify as Black, Latinx, or Pacific Islander. Those are the communities that have shown the highest rates of COVID and are impacted the most by the economic fallout of COVID. And so thinking of situations where our doulas are single parents. Our doulas live with elders. Our doulas have multiple jobs. That if they get sick, it's not just being out of SisterWeb work, potentially without health benefits. It's also being out of their other job.” (Leader)

In addition, being able to switch out with other doulas during long births is a key component of SisterWeb's cohort model^26^; leaders advocated to ensure doulas would be able to do so, since hospitals were not allowing support people to take shifts or return after leaving. Leaders communicated with provider “champions” at hospitals established through the Champion Dyad Initiative, which allows for bidirectional feedback between SisterWeb and birth sites.^17^ After trainings that covered providing support amid COVID-19 and how to properly use PPE, SisterWeb doulas returned to attending births in person in July 2020. Prenatal and postpartum visits remained mostly virtual.

### Adapting to changing hospital policies and care

Throughout the pandemic, hospitals' COVID-19 protocols varied. Protocols changed frequently, which created confusion among hospital staff, doulas, and clients and impacted pregnancy-related care and birth. One doula obtained permission from the hospital to attend a birth, only to be turned away when she arrived; she described a nurse manager witnessing her attempts to reach a client:

“She heard the whole entire thing of me trying to get in, heard my client on the phone crying, and just would not budge with trying to let me in. At that point, I didn't even know what I was going to do when I got in there. I was just going to, like, hold her hand. But she just needed extra support. And they were not trying to hear it.” (Doula)

Individual providers enforced rules differently, leading to inconsistencies. Issues also arose when there were lapses in communication between providers and hospital security. Doulas described not being allowed past security, even when providers gave them permission to attend births in person. Doulas witnessed how navigating hospital rules “put a strain” (Doula) on clients and affected their health care experiences. Despite the importance of postpartum doula support, certain hospitals did not allow doulas to follow clients from Labor and Delivery (L&D) to the Postpartum unit, where some clients reported experiencing mistreatment.

Although some doulas said certain hospitals' Postpartum units were stricter than L&D units prepandemic, one doula described an instance in which a nurse told a postpartum client she could not reheat food due to COVID-19 protocols. The nurse later yelled at the client over a miscommunication about whether the client could order a meal through a food-delivery app and questioned whether she could pay for it. When the doula visited the same client in the hospital, she described witnessing how nurses enforced rules differently, impacting the client's intended feeding plan:

“Even I was noticing some passive-aggressive stuff while I was in there. The baby was getting donor milk. Again, it was OK with the nurse who brought it in with her, with the nurse at the desk. But then when [another nurse] found out she had [the donor milk], it was like, ‘Oh, it has to go through x, y, and z channels before you can get approved.’ Takes the milk out of the room. And it's like, at this point, what are you going to do? The baby already started drinking from it. You can't use this on somebody else.” (Doula)

### Adapting to shifting modes of client support

Over time, doulas adapted to virtually supporting clients and eventually transitioned to a hybrid model. Doulas said they refined certain skills, such as holding space virtually, asking probing questions, and becoming more active listeners. However, doulas worried virtual support impacted their ability to establish trust with clients, provide adequate emotional support, advocate for them in hospital settings, and watch for signs of postpartum complications. Meeting in person is particularly important to the communities SisterWeb serves:

“We make do, but it's very hard to not see people in person. Especially for our clients that have experienced trauma, and then are generally high stress, being in person really helps them be grounded and embodied.” (Doula)

Doulas began connecting with clients more often through phone and text but reduced the amount of time they spent on each virtual visit. Frequent communication helped doulas build trust with clients in a virtual environment and let clients know they had support during a stressful time.

Once a hybrid model was implemented, with in-person attendance of births and virtual support during the prenatal period, it meant that doulas were not meeting clients face-to-face until the birth, “which is potentially the single most intimate and intense experience that they've had in their life” (Doula).

Although SisterWeb permitted doulas to meet in person for all visits in 2022, doulas said clients preferred a mix of in-person and virtual support for prenatal and postpartum visits. After experiencing a few no-shows for in-person visits, one doula realized her clients were more comfortable meeting virtually:

“I don't know if it's like a mixture of fear, or just complacent, or just familiarity. I think all of our mentality has been, ‘Give me space.’ And even being vaccinated has its confusion as well, because it's like, I can say all day, ‘Look, I'm super vaxxed.’ But it still doesn't negate the fact that I can still catch it and give it to people.” (Doula)

### Adapting to supporting doulas' well-being

Doula mentors filled an important role as the pandemic began to wear on doulas' emotional and mental health. One mentor said doulas must be provided emotional support so they are better equipped to support marginalized clients who have experienced trauma. Through her mentorship role, she helped doulas process their work, which became even more taxing in 2020:

“So then, added the layer of COVID, and then it's been a very, very intense year. And I think I've had an opportunity to really sink in and feel the tears and the pain.” (Mentor)

Mentors listened to doulas' fears related to COVID-19 and heard them express frustrations when they could not be physically present to “help moms feel safe in the hospital” (Mentor). When probed about whether she felt equipped to provide the level of emotional support needed during the pandemic, a mentor noted that she was not trained as a counselor or therapist. The mentor described how she approached supporting mentees during a period when they were not allowed to provide in-person support:

“It's always hard to hear the frustration of someone wanting to do more and not being able to because I can relate to that so greatly. I think birth work is very political. You're wanting to help someone feel empowered and safe in their body and make sure they're treated with respect. So when you're not there to make sure of that, you almost feel like, well, what am I even doing? Am I even helping? So I think just reminding [the doulas] that they were still so useful in more ways than they could imagine. I wasn't super equipped. I was just trying my best as a human.” (Mentor)

## Discussion

Our study documented how a community-based doula organization that employs and serves Black, Pacific Islander, and Latina/o/x people in San Francisco adapted to the COVID-19 pandemic over 2 years. By shifting to virtual support until doulas were onboarded as hourly, benefitted employees, SisterWeb leaders chose to prioritize the health, safety, and financial stability of staff members, who were members of the same communities of color that were disproportionately impacted by COVID-19. Through SisterWeb's mentorship program, doulas had already established trusting relationships with mentors before the pandemic. As the pandemic began to impact doulas' mental and emotional health, mentors provided critical support. Doulas also described how COVID-19 policies negatively impacted clients' health care experiences.

SisterWeb leaders utilized relationships established through the Champion Dyad Initiative to call out inequitable hospital policies, such as barring doulas, who have other jobs and caretaking responsibilities, from switching with other doulas so clients could receive continuous support. Despite SisterWeb's institutional relationship with partners at hospitals, doulas encountered hurdles when trying to support clients of color. Our findings suggest that COVID-19 hospital policies limited the extent of support that SisterWeb doulas could offer their clients. Although some hospital administrators became increasingly open to doula presence in L&D units throughout the pandemic, they did not always extend the same policies to Postpartum units. Hospital administrators should be mindful of the importance of a doula's role during the postpartum period.

In addition, we found that once SisterWeb allowed in-person support, doulas adjusted their model of care according to client preference for virtual, in-person, or hybrid support. This could have future implications for the modalities of doula support. As more states begin to implement Medicaid coverage of doula care, policymakers should incorporate telehealth options into doula benefits to meet client needs.

This study adds to a growing body of literature describing doulas' experiences during the pandemic. Unlike most studies of birth workers in the United States during the pandemic, our study provides an organizational perspective. Similar to our findings, other studies found doulas grappled with the loss of human connection with clients but adapted to virtual support.^4,11,23,24^ Doulas and their clients also faced inconsistent hospital policies that excluded support people and contributed to negative health care experiences during a time when birthing people faced “significant uncertainty and anxiety.”^10,12,21,30^ For example, doulas in our study described how hospital protocols varied by site and were enforced differently by providers, which may have impacted clients' pregnancy-related care. Future research should examine the long-term effects of limiting the number of support people during public health emergencies and how health care professionals can enforce policies more equitably.

Strengths of this study include longitudinal qualitative data collection, allowing us to capture how SisterWeb staff adapted to multiple COVID-19 waves and shifts in public health guidance and hospital restrictions. Our study also included perspectives from leaders, mentors, and doulas, allowing us to gain a comprehensive understanding of the organization's adaptions to COVID-19. Limitations of our analysis include a lack of data on doulas serving Latinx/a/o families due to funding limitations.

Our analysis is specific to one community doula organization in the Bay Area, the first region in the United States to issue shelter-in-place restrictions.^31^ Although we interviewed doula clients before and after their births during the process evaluation, a majority of data collection occurred before the pandemic. Therefore, we did not include client data in this analysis, and we cannot make inferences about clients' experiences of SisterWeb's COVID-19 adaptations. However, SisterWeb clients who received doula support during the pandemic reported positive experiences.^32^

SisterWeb employs and serves populations that were disproportionately impacted by the COVID-19 pandemic. Our findings suggest that the confluence of COVID-19, systemic and structural racism, and SisterWeb's commitment to acting within organizational values made it difficult for SisterWeb to simultaneously support doulas and clients as envisioned. When shelter-in-place began in San Francisco in March 2020,^31^ public health ordinances related to COVID-19 and subsequent hospital policies limited in-person birth support. At the time, most SisterWeb doulas were independent contractors who did not receive health insurance or paid sick leave through SisterWeb. Notably, one of the challenges doulas, who often work as contractors, face is low pay and lack of job benefits.^33^

As policies changed and more doulas returned to hospital-based births, SisterWeb leaders waited until doulas were onboarded as hourly employees eligible for benefits. Our findings suggest that SisterWeb's initial lack of a sustainable funding structure ultimately hampered the organization's ability to serve clients with limited support systems. However, by transitioning to a more dignified compensation model, SisterWeb adhered to its belief that community doulas deserve sustainable employment options.^34^ We also found that emotional support for doulas, who witness racism and obstetrical violence, is vital to their work. Funders should consider the need for health benefits and access to mental health services for doulas and staff members when funding community-based doula programs.

## Abbreviations Used

KBC: Kindred Birth Companions
L&D: Labor and Delivery
RAP: Rapid Assessment Process
